# Yes-Associated Protein 1 Plays Major Roles in Pancreatic Stellate Cell Activation and Fibroinflammatory Responses

**DOI:** 10.3389/fphys.2019.01467

**Published:** 2019-12-03

**Authors:** Cheng Hu, Jiayue Yang, Hsin-Yuan Su, Richard T. Waldron, Mengmeng Zhi, Ling Li, Qing Xia, Stephen J. Pandol, Aurelia Lugea

**Affiliations:** ^1^Department and Laboratory of Integrated Traditional Chinese and Western Medicine, Sichuan Provincial Pancreatitis Centre and West China-Liverpool Biomedical Research Centre, West China Hospital, Sichuan University, Chengdu, China; ^2^Departments of Medicine and Biomedical Sciences, Cedars-Sinai Medical Center, Los Angeles, CA, United States; ^3^Department of Endocrinology, Zhongda Hospital Southeast University, Nanjing, China; ^4^Department of Medicine, David Geffen School of Medicine at UCLA, Los Angeles, CA, United States

**Keywords:** pancreatic stellate cells, pancreatitis, pancreatic cancer, yes-associated protein 1, fibrosis

## Abstract

**Background:** Yes-associated protein 1 (YAP), a transcriptional co-activator and major effector of the Hippo pathway, regulates cell differentiation and morphology in many cell types and supports aberrant tumor growth. Recent studies showed that YAP is expressed in pancreas tissues in pancreatic ductal adenocarcinoma (PDAC) patients and experimental models of PDAC, with YAP largely found in cancer cells and pancreatic stellate cells (PaSC) in the stroma.

**Methods and Results:** We studied here the role of YAP in the activated phenotype of PaSC. We found that YAP is expressed at low levels in normal mouse pancreas, but protein levels significantly increased after pancreas inflammatory damage induced by repeated cerulein administration in wild-type mice or upon initiation of neoplastic transformation of the pancreas parenchyma in Ptf1-Cre;LSL-Kras^G12D/+^ (KC) mice. In these animal models, YAP upregulation occurred in parallel with activation and proliferation of PaSC. Consistent with these findings, we found robust YAP expression in culture-activated mouse and human PaSC but not in quiescent, freshly isolated cells. Fully activated PaSC isolated from KC mice or PDAC patient tissues exhibited robust nuclear YAP suggesting YAP transcriptional activity. Agents that induce quiescence such as the Bromodomain and Extra-Terminal (BET) inhibitor iBET151 and the p38 MAPK inhibitor SB203580 reduced YAP levels in PaSC. Stimulation of PaSC with the potent mitogen PDGF elicited marked YAP Ser127 phosphorylation. However, unexpectedly, this effect did not diminish YAP nuclear localization, suggesting that YAP phosphorylation at this site does not govern YAP cellular localization in PaSC. siRNA-mediated knockdown of YAP reduced PDGF-induced PaSC expansion in culture and blunted the persistent activation of Akt and ERK elicited by PDGF stimulation, supporting a role for YAP in PDGF-induced cell growth. YAP knockdown also blunted fibroinflammatory gene expression responses both in unstimulated and transforming growth factor beta 1 (TGFβ1)-stimulated PaSC.

**Conclusion:** Our data suggest a central role for YAP in sustaining the activated phenotype and fibroinflammatory responses in PaSC. Moreover, our findings indicate that a complex crosstalk between YAP, TGFβ1, and PDGF pathways regulates PaSC activity and growth.

## Introduction

Acute (AP), recurrent acute (RAP), and chronic pancreatitis CP are inflammatory disorders of the exocrine pancreas associated with significant morbidity, a high rate of hospitalizations and mortality (Peery et al., 2012; Lew et al., 2017). Further, CP of diverse etiologies significantly increases the risk for pancreatic ductal adenocarcinoma (PDAC) (Lew et al., 2017; Yang and Forsmark, 2017). In this respect, epidemiological studies indicate that CP patients at 5 years after diagnosis have a nearly eight-fold increased risk of acquiring PDAC (Kirkegard et al., 2017). Currently, there are no effective treatments to prevent or attenuate pancreatitis or to prevent RAP and CP from progressing to PDAC, as occurs in some patients.

The precise mechanisms of initiation and progression of CP are unclear. CP is considered to result from unresolved, recurrent fibroinflammation and is characterized by extensive loss of the normal exocrine parenchyma, widespread fibrosis and inflammation, and impairment of both exocrine and endocrine pancreatic functions (Majumder and Chari, 2016; Yang and Forsmark, 2017; Gukovskaya et al., 2019). Accumulating evidence supports the concept that pancreatitis is initiated in the pancreatic acinar cell, the main cell type in the exocrine parenchyma, with stresses causing cellular dysfunction that triggers activation of pro-inflammatory signaling, activation of neighboring quiescent pancreatic stellate cells (PaSC), and immune cell infiltration into the pancreas (Gukovskaya et al., 2019).

PaSC are mesenchymal cells residing in the pancreas. In the normal pancreas, quiescent, non-proliferative PaSC reside in the periacinar space and are estimated to represent about 5–7% of the exocrine parenchymal cells (Omary et al., 2007). Upon pancreas damage, or during the neoplastic transformation of acinar and ductal cells, PaSC acquire a myofibroblast phenotype, a process termed “activation” (Omary et al., 2007; Apte et al., 2013). Characteristics of activated PaSC include high proliferation and growth rates; and expression of marker proteins such as alpha-smooth muscle actin (αSMA), a contractile cytoskeleton protein organized in stress fibers that confer mechanical tension, and cadherin 11 (CDH11), which regulates extracellular matrix (ECM) synthesis and matrix properties (Row et al., 2016). Activated PaSC produce and secrete large quantities of various ECM proteins, cytokines, and growth factors that regulate matrix physical properties, ECM remodeling, and fibrogenesis, as well as immune cell infiltration and inflammatory responses in RAP, CP, and PDAC tumors (Apte et al., 2012, 2013; Erkan et al., 2012; Pandol et al., 2012; Dawson et al., 2013; Chang et al., 2017). Intensive research has demonstrated that PaSC exhibit great plasticity that allows reprogramming in response to multiple inputs from the matrix, neighboring acinar, cancer and immune cells, and local and systemic factors (Ohlund et al., 2017). Recent work has also shown that PaSC play pivotal roles in other pancreatic disorders including diabetes (Yang et al., 2016). However, despite the critical role of PaSC in pancreas pathobiology, the mechanisms governing their activation, growth, and phenotypic plasticity remain unclear.

Recent studies indicate that Yes-associated protein 1 (YAP) and its homolog WWTR1/TAZ are expressed in pancreatic cells including PaSC during tissue remodeling and PDAC initiation (Morvaridi et al., 2015; Rozengurt et al., 2018; Ansari et al., 2019; Eibl and Rozengurt, 2019). YAP and TAZ are transcriptional regulators that modulate cell proliferation, migration, and apoptosis in developing organs and adult tissues and support aberrant cell growth in many cancers (Eibl and Rozengurt, 2019). By virtue of their sensitivity to mechanical cues such as extracellular matrix rigidity, YAP/TAZ act as mechano-transducers coupling cell-cell and cell-matrix signals with gene expression (Dupont et al., 2011). YAP is found in the cytoplasm and in the nucleus where it interacts with TEF-1/TEC1/abaA (TEA) domain 1-4 (TEAD1-4) and other transcription factors including SMADs involved in cell proliferation, matrix remodeling, and apoptosis (Zheng and Pan, 2019). YAP functions are regulated in a cell type- and context-dependent manner. In myofibroblasts and other cell types, YAP expression and subcellular location are regulated by the Hippo pathway, matrix rigidity, and the mechanical properties of the cellular cytoskeleton (Dupont et al., 2011). Indeed, increased matrix stiffness and high cytoskeletal tension promote YAP nuclear accumulation and transcriptional activity in cancer associated myofibroblasts (Calvo et al., 2013). Recent studies indicate that YAP regulates activation in liver of pro-fibrotic hepatic stellate cells (Mannaerts et al., 2015), a cell type phenotypically similar to PaSC, but the role of YAP in PaSC activation and fibroinflammatory responses has not been defined.

Here, we examined YAP expression and activity during RAP and CP and in quiescent and activated PaSC derived from mouse and human pancreas. We also investigated the effects of agents that we found promote PaSC quiescence, and siRNA-mediated YAP knockdown, on PaSC growth signaling, fibroinflammatory responses, and signaling crosstalk between YAP and two well characterized PaSC activators, platelet-derived growth factor (PDGF) and transforming growth factor beta 1 (TGFβ1).

## Materials and Methods

### Antibodies and Chemicals

The following antibodies were used for Western blotting (WB) and/or immunofluorescence (IF): AKT (#4691), phospho-AKT (Ser473; #4060), Cadherin11 (CDH11; #4442S), GAPDH (#5174S), CK19 (#12434), p44/42 MAPK (#9102), LATS1 (#3477), phospho-LATS1 (Thr1079; #8654), phospho-p44/42 MAPK (Thr202/Tyr204; #9101), phospho-p70S6 Kinase (Thr389; #9234), SMAD2/3 (#8685), phospho SMAD2 (Ser465/467)/SMAD3(Ser423/425); #8828), YAP (#14074 for WB and IF, #4912 for WB), phospho-YAP (Ser127; #13008), YAP/TAZ (#8418), Caspase-3 (#14220), and corresponding HRP-linked secondary antibodies were from Cell Signaling Technology (Danvers, MA). Antibodies directed against αSMA (#A2547), P4HA2 (#SAB1100773), and β-actin (#A1978) were purchased from Sigma-Aldrich (St. Louis, MO), against GFAP (#ab68428) from Abcam (Cambridge, MA), and against PDGFR-β (#sc-432) from Santa Cruz Biotechnology (Dallas, TX).

Recombinant rat PDGF-BB (PDGF; #520-BB-050), recombinant mouse TGFβ1 (#7666-MB-005), and recombinant human TGFβ1 (#240-B-002) were from R&D Systems, Inc. (Minneapolis, MN). iBet151 (#SML0666) was purchased from Sigma-Aldrich (St. Louis, MO); U0126 (#662005), LY294002 (#440202) and SB203580 (#559389) from MilliporeSigma (Burlington, MA). SuperSignal™ West Pico (or Femto) Chemiluminescent Substrate reagent, 4′,6-diamidino-2-phenylindole (DAPI), ProLong Gold antifade mounting medium, and fluorescence-conjugated secondary antibodies were from ThermoFisher Scientific (Waltham, MA).

Tissue digestion for primary PaSC isolation was performed using Pronase (Roche, #10165921001), Collagenase P (Roche, #11213857001), DNase I (#10104159001) and bovine serum albumin fraction V (BSA; Roche, #3116956001), all obtained from Sigma-Aldrich. Density gradients for PaSC separation were prepared using Nycodenz (#AN1002423; Accurate Chemical & Scientific Corp; Westbury, NY) and Gey’s balanced salt solution (GBSS; #G9779; Sigma-Aldrich). Cell culture DMEM/F12 medium (#11330-032), L-Glutamine (#25030-081) were from ThermoFisher Scientific; antibiotics/antimycotics (1% Penicillin–Streptomycin; #25030-081) and fetal bovine serum (FBS; #FB11) were from Omega Scientific (Tarzana, CA). All chemicals and kits were used according to the manufacturer’s recommendations, unless otherwise indicated.

### Assessment of Yes-Associated Protein 1 Levels in Pancreatic Tissues From Mice Subjected to Cerulein-Induced Pancreatitis

C57BL/6 male mice (Envigo, Placentia, CA) were subjected to repeated episodes of acute cerulein pancreatitis starting at 6–7 weeks of age. Each acute pancreatitis (AP) episode consists of 7 hourly intraperitoneal injections of saline or 50 μg/kg cerulein (Lugea et al., 2006). Recurrent AP (RAP) was characterized in mice subjected to two episodes of cerulein AP, the first one at day 1 (d1) and the second at day 3 (d3). In this RAP model, mice were sacrificed during the acute phase of pancreatitis (1 h after the last cerulein injection, at d1 and d3) and during the recovery phase (at d5). Chronic pancreatitis (CP) was induced by repeated cerulein AP episodes (twice a week for 4 weeks; total eight episodes), and mice were sacrificed 4 days after the last AP episode. At sacrificed, pancreatic tissues were collected and snap-frozen for subsequent Western blotting analysis (as indicated below) or formalin-fixed for histological assessment of pancreatitis severity, PaSC activation and immunofluorescence analysis of YAP and PaSC activation markers αSMA, Cadherin 11 (CDH11) and PDGF receptor beta (PDGFRβ) expression.

Mice were fed standard chow diet with free access to clean drinking water and maintained at controlled temperature (19–22°C) and 12:12-h light/dark cycle during the duration of the study. Animal studies were approved by the Institutional Animal Care and Use Committee at Cedars-Sinai Medical Center (Los Angeles, CA) in accordance with the NIH Guide for the Care and Use of Laboratory Animals.

### Assessment of Yes-Associated Protein 1 Levels in Kras^G12D^ Mice

Wild-type (WT) and Ptf1-Cre;LSL-Kras^G12D/+^ (KC) mice, a genetically engineered mouse model (GEMM) used to study pancreatic ductal adenocarcinoma (PDAC), were generated by breeding as previously described in our studies (Dawson et al., 2013; Chang et al., 2017). Mice were fed standard chow diet and maintained in standard housing conditions [controlled temperature (19–22°C) and 12:12-h light/dark cycle] during the duration of the study. Mice were sacrificed at 3 months and pancreas tissues harvested for histological and Western blotting protein analysis of YAP and PaSC markers as described before (Morvaridi et al., 2015; Chang et al., 2017). Animal studies were approved by the Institutional Animal Care and Use Committee at Cedars-Sinai Medical Center (Los Angeles, CA) in accordance with the NIH Guide for the Care and Use of Laboratory Animals.

### Immunofluorescence Analysis of Yes-Associated Protein 1 and Alpha-Smooth Muscle Actin in Mouse Pancreatic Tissues and Cultured Pancreatic Stellate Cells

Formalin-fixed, paraffin-embedded (FFPE) mouse pancreatic tissues were obtained from WT mice subjected to RAP or CP as described above. Four micrometer tissue sections were stained by IF using specific antibodies against YAP (#14074, Cell Signaling Technology) and αSMA (#A2547, Sigma-Aldrich; marker of activated PaSC). Alexa Fluor 488 or Alexa Fluor 594 was used as conjugated secondary antibodies, and 4′6′-diamidino-2-phenylindole (DAPI) as nuclear counterstain. Digitalized images were captured using a Leica TCS SP5 spectral confocal microscope (Leica Microsystems, IL) and analyzed with the Leica Application Suite Advanced Fluorescence Lite 2.6.0 software (LAS-AF-lite; Leica Microsystems Inc., Buffalo Grove, IL) with the assistance of the Cedars-Sinai Medical Center (Los Angeles, CA) Imaging Core. *Similar IF procedures were used to assess YAP and αSMA in cultured PaSC*.

Cellular localization of YAP in cultured PaSC was assessed using Image J by measuring the ratio of nuclear-to-cytoplasmic fluorescence intensity, expressed in each region as integrated intensity per unit area. In these studies, YAP quantification was performed in 70–90 cells from three to five representative microscopic fields.

### Pancreatic Stellate Cells Isolation and Culture

Primary mouse PaSC (mPaSC) were obtained from wild-type and KC pancreas tissues as previously described (Pandol et al., 2012; Su et al., 2016; Yang et al., 2016). Briefly, pancreata from one to two mice were excised, minced, and digested in GBSS containing a mixture of pronase, collagenase P and DNase I. The cell suspension was filtered through a 100 μm nylon cell strainer and washed in GBSS supplemented with 0.3% BSA. Then, PaSC were separated by Nycodenz density gradient centrifugation (20 min at 1,400 g). Quiescent PaSC were collected from a fuzzy band at the interface near the top of the gradient and expanded in culture up to passage 2. Myofibroblast-like activated mPaSC were characterized by the presence of αSMA stress fibers, high production of fibronectin, and reduced expression of the quiescent marker GFAP.

Immortalized mouse PaSC (imPaSC) were initially obtained from Dr. Raul Urrutia and characterized in our previous studies (Su et al., 2016; Yang et al., 2016). Human primary PaSC (hPaSC) were obtained from pancreatic cadaveric tissues from organ donors or PDAC surgical resections. Briefly, cadaveric pancreata were digested at City of Hope (Duarte, CA) to isolate islets for clinical transplantation to diabetic patients as described (Song et al., 2015; Lugea et al., 2017). Remnant pancreatic cells after islet isolation (mainly pancreatic acinar, ductal, and PaSC) were further processed at the Cedars-Sinai Medical Center (Los Angeles, CA) to isolate hPaSC following similar procedures indicated above for primary mPaSC. hPaSC from PDAC tissues were obtained by the outgrowth method as previously described (Bachem et al., 2005; Lugea et al., 2006). The studies were performed in accordance with regulations and IRB protocols approved by the Institutional Review Board at Cedars-Sinai Medical Center (Pro31101 and Pro32114).

Primary mPaSC and hPaSC, and imPaSC were grown in DMEM/F12 media supplemented with 10% FBS, 2 mM L-glutamine, and antibiotics/antimycotics, in a humidified 5% CO_2_ atmosphere. For experiments, cells were seeded in 60-mm tissue culture dishes in DMEM/F12 and starved in serum-free medium for 12 h, and then treated with the experimental agents at 60–80% confluency, unless otherwise stated.

### Knockdown of Yes-Associated Protein 1 *Via* siRNA Transfection

siRNA targeted against mouse YAP1 mRNA were obtained from ThermoFisher Scientific (#4390771Waltham, MA). Control transfections were carried out with Silencer Select Negative Control No. 1 (#4390843, ThermoFisher Scientific). For siRNA transfection, imPaSC (1.5 × 10^5^ cells/plate) were cultured in 60 mm plates until 60% confluence. Silencer nontargeting negative control (10 nmol/L; Mock transfection) or YAP siRNA (10 nmol/L) were mixed with Lipofectamine RNAiMAX (#13778075 ThermoFisher Scientific) according to the manufacturer’s recommendations and added to the cells. After transfection, imPaSC were cultured in DMEM/F12 medium containing 10% FBS for 24 h and then used for experiments.

### Western Blot Analysis

Cells or tissues were homogenized in RIPA buffer containing 50 mmol/L Tris (pH 7.4), 150 mmol/L NaCl, 1% deoxycholic acid, 1% Triton X-100, 0.1% SDS, and a mix of protease and phosphatase inhibitors (Roche Applied Science, Basel, Switzerland). Protein extracts were resolved by SDS-PAGE for immunoblot analysis. The primary and horseradish peroxidase-conjugated secondary antibodies used here are indicated in the Antibodies and chemicals section. Immunoreactive bands were visualized by chemiluminescence (ThermoFisher Scientific) and densitometrically quantified using the PXi 6 Touch Imaging System (Syngene). To estimate protein levels, optical density values in each blot were expressed relative to those of the loading control (ERK, β-actin or GAPDH).

### RNA Analysis by Qualitative Polymerase Chain Recation

Cellular RNA from quiescent and culture-activated PaSC was extracted using the RNeasy^®^ Plus Mini Kit (#74034; Qiagen, Germantown, MD). Reverse transcription was performed with the iScript Reverse Transcription Supermix (#170-8,840; Bio-Rad, Hercules, CA) using 1 μg of total RNA, and the synthesized cDNA samples were used as templates for quantitative real-time PCR (qPCR) analysis. Kits were used according to the manufacturer’s instructions. All reactions were performed using the Bio-Rad CFX ConnectTM Real-Time PCR Detection System and the amplifications were done with the iTaq^™^ Universal SYBR^®^ Green Supermix (Bio-Rad). The gene-specific primers used are listed in Table 1. Relative transcript levels were calculated using the comparative 2^−ΔΔCt^ method and normalized to the housekeeping gene, 18S rRNA.

**Table 1:** Primer sequences for qRT-PCR.

Target	Forward (5′ to 3′)	Reverse (5′ to 3′)
mActa2 (αSMA)	GTTCAGTGGTGCCTCTGTCA	ACTGGGACGACATGGAAAAG
mCol1a1	TAGGCCATTGTGTATGCAGC	ACATGTTCAGCTTTGTGGACC
mCcn1 (Cyr61)	TGCTGTAAGGTCTGCGCTAA	GGTCTGCCTTCTGACTGAGC
mCcn2 (Ctgf)	CTGCCTACCGACTGGAAGAC	CATTGGTAACTCGGGTGGAG
mGfap	AAGAAAACCGCATCACCA	ACAACTTGTATTGTGAGCCT
mHgf	CTTTTTGCCTTCGAGCTATC	GGTCATGCATTCAACTTCTG
mIl6	ACCAGAGGAAATTTTCAATAGGC	TGATGCACTTGCAGAAAACA
mMmp3	AGCCTTGGCTGAGTGGTAGA	CGATGATGAACGATGGACAG
mSerpine1 (PAI-1)	ATCCTGCCTAAGTTCTCTCTG	ATTGTCTCTGTCGGGTTGTG
mTgfb1	CAACCCAGGTCCTTCCTAAA	GGAGAGCCCTGGATACCAAC
mYap1	GCTGCAGCAGTTACAGATGG	GCGCAGAGCTAATTCCTGAC
hACTA2 (αSMA)	CCAGAGCCATTGTCACACAC	CAGCCAAGCACTGTCAGG
hWWTR1 (TAZ)	GTCCTACGACGTGACCGAC	CACGAGATTTGGCTGGGGA
hYAP1	TAGCCCTGCGTAGCCAGTTA	TCATGCTTAGTCCACTGTCTGT
18S rRNA	AGTCCCTGCCCTTTGTACACA	CGATCCGAGGGCCTCACTA

### MTT Assay

MTT (Thiazolyl Blue Tetrazolium Bromide) assay was used as indicator of cellular metabolic activity and cell proliferation. PaSC were seeded in 24-well plates at 1 × 10^4^ cells per well. After the indicated treatments, MTT was added to the culture medium to a final concentration of 0.5 mg/ml, and cells were then incubated for 3 h at 37°C in a 5% CO_2_ incubator. After removing medium, DMSO was added to dissolve the formazan product. Absorbance was measured at 595 nm using a spectrophotometric plate reader (SpectraMax M3, Molecular Devices, San Jose, CA).

### Cell Death

Cell necrosis was assessed by PI uptake. Briefly, cells were treated with the experimental reagents for up to 72 h, and then labeled with PI (2 μg/ml medium) for 10 min. Both attached and floating cells were then collected, washed with PBS to remove excess PI, and lysed in RIPA buffer [50 mmol/L Tris (pH 7.4), 150 mmol/L NaCl, 0.25% deoxycholic acid, 1% Triton X-100, 0.1% SDS containing a mixture of protease and phosphatase inhibitors (Roche Applied Science, Basel, Switzerland)]. PI fluorescence was measured by fluorometry at 535_ex_/617_em_ nm, and values normalized to those of total protein concentration in cell lysates. Caspase 3-dependent apoptosis was assessed by measuring levels of total and cleaved Caspase 3 by Western blotting.

### Statistical Analyses

All experiments were performed in triplicate unless otherwise stated. Data are presented as mean ± SD or mean ± SEM. Data were subjected to analysis of variance (ANOVA) followed by Tukey’s *post hoc* test, and two tailed Student *t*-test for comparison between 2 groups. Differences between treatments were considered to be statistically significant at *p <* 0.05 as indicated in the figure legends.

## Results

### Yes-Associated Protein 1 Upregulation During Experimental Pancreatitis and Pancreatic Ductal Adenocarcinoma Progression Parallels Pancreatic Stellate Cells Activation

Previous studies indicate that effectors of the Hippo signaling system including TEAD1 and its transcriptional activator YAP are integral components of the embryonic development of human pancreas (Cebola et al., 2015) and its expression is recapitulated during progression of pancreatic disorders (Kapoor et al., 2014; Zhang et al., 2014; Morvaridi et al., 2015; Hao et al., 2017). Histological analysis of pancreatic tissues from PDAC patients and murine models of pancreatic cancer shows that YAP is upregulated in precancerous lesions (Pancreatic Intraepithelial Neoplasia, PanIN) and cancer cells as well as in activated PaSC in the stroma (Kapoor et al., 2014; Zhang et al., 2014; Morvaridi et al., 2015; Hao et al., 2017). These findings highlight the therapeutic potential of targeting YAP signaling to prevent PaSC activation and fibrosis in PDAC and CP, two pancreatic disorders showing extensive desmoplastic reaction.

To evaluate the relationship between YAP and PaSC activation during pancreatitis, we used experimental mouse models of AP, RAP, and CP. AP was induced by administration of high doses of the cholecystokinin analogue cerulein (50 μg/kg; 7 hourly intraperitoneal injections) and tissues collected during the acute phase (1 h after the last cerulein injection). RAP was induced by two episodes of AP (at days 1 and 3), and mice sacrificed during the acute phase (1 h after the last cerulein injection; day 3) and during the recovery phase (day 5), when pancreas undergo extensive remodeling. CP was induced by repeated episodes of cerulein AP (two episodes per week, for 4 weeks) and mice sacrificed 4 days after the last episode, when marked PaSC activation is evident (Lugea et al., 2006). Supplementary Figure S1A shows representative H&E micrographs of pancreas histology in control- and cerulein-treated mice. As shown, repeated cerulein AP leads to loss of normal exocrine parenchyma, acinar cell death, inflammatory cell infiltration and stromal expansion.

**Figure 1:**
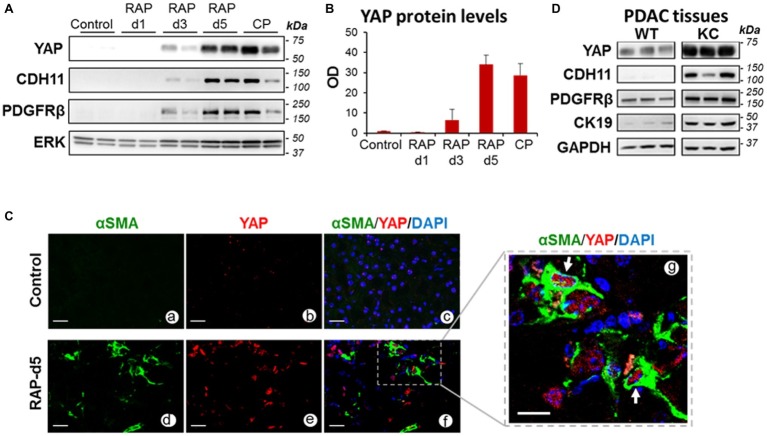
YAP levels increase in pancreas during parenchyma remodeling in pancreatic disease. **(A)** Representative immunoblots showing protein levels of YAP and the PaSC markers Cadherin 11 (CDH11) and the PDGF receptor PDGFRβ in pancreas of mice subjected to repeated episodes of cerulein acute pancreatitis: *RAP*; two episodes, 1 day apart; *CP*, chronic pancreatitis, two episodes per week for 4 weeks. As indicated in the “Methods” section, mice were sacrificed during the acute phase of pancreatitis (**d1**, day 1, one AP episode; **d3**, day 3, two AP episodes), 2 days after two AP episodes (**d5**) or 4 days after the last episodes in the **CP** model. ERK1/2 was used as loading control. Each lane shows data from an individual mouse; two mice per group are shown. Data are representative of five mice per group. **(B)** Graph shows quantitation of optical density of YAP bands in immunoblot shown in panel **A** relative to those of ERK1/2. Data are mean ± SEM, *n* = 5 mice/group. **(C)** Paraffin-embedded pancreatic tissues obtained from mice subjected to two episodes of AP (days 1 and 3) and sacrificed at day 5 (**RAP-d5**) or saline-treated mice (**Control**) were analyzed for the PaSC marker αSMA and YAP by immunofluorescence. Nuclei were counterstained with DAPI. Panels **a–f** show representative immunofluorescence staining; Scale bar = 25 μm. Dotted box in panel **f** shows αSMA-positive PaSC displaying YAP staining in the nucleus; the dotted area is shown higher magnified in panel **g**. Arrows in panel **g** show positive YAP nuclei. Scale bar = 10 μm. Control tissues (panels **a–c**) do not exhibit activated PaSC or significant YAP staining. Data are representative of three mice per group. *Representative H&E stained images from control and RAP-5d mice pancreas are shown in* Supplementary Figure S1A. **(D)** Representative immunoblots showing protein levels of YAP, the PaSC markers CDH11 and PDGFRβ, the ductal marker Cytokeratin 19 (CK19) in pancreas of 2-month-old wild-type (**WT**) and Ptf1-Cre;LSL-Kras^G12D/+^ (**KC**) mice. GAPDH was used as loading control. Each lane shows data from an individual mouse; three mice per group are shown. *Representative photomicrographs from KC mouse pancreas showing a-SMA positive PaSC are included in* Supplementary Figure S1B.

In pancreas of control (saline-treated) mice or mice subjected to one episode of AP (day 1), YAP protein expression was negligible (Figures 1A–C). YAP levels increased moderately after the second episode of AP (day 3) but markedly 2 days after RAP induction (day 5) and remained elevated during CP (Figures 1A,B). YAP upregulation was concomitant with upregulation of the PaSC markers CDH11 and PDGFRβ (Figure 1A). Moreover, αSMA positive, activated PaSC in RAP (Figure 1C) and CP pancreas (Morvaridi et al., 2015) exhibited marked YAP nuclear staining, suggesting YAP transcriptional activity in these cells.

As our group previously reported (Morvaridi et al., 2015), YAP was markedly upregulated in pancreas of KC mice. Illustrated in Supplementary Figure S1B, pancreas tissues of KC mice display abundant PanIN surrounded by stroma enriched in αSMA-positive PaSC. In these mice, pancreatic levels of YAP increased in parallel with PanIN and stromal progression. Figure 1D shows significant upregulation of YAP, the PaSC markers PDGFRβ and CDH11, and the ductal marker cytokeratin 19 (CK19) in pancreatic extracts of KC mice compared with wild-type mice.

### Yes-Associated Protein 1 Is Expressed in Activated but Not Quiescent Pancreatic Stellate Cells

**Figure 2:**
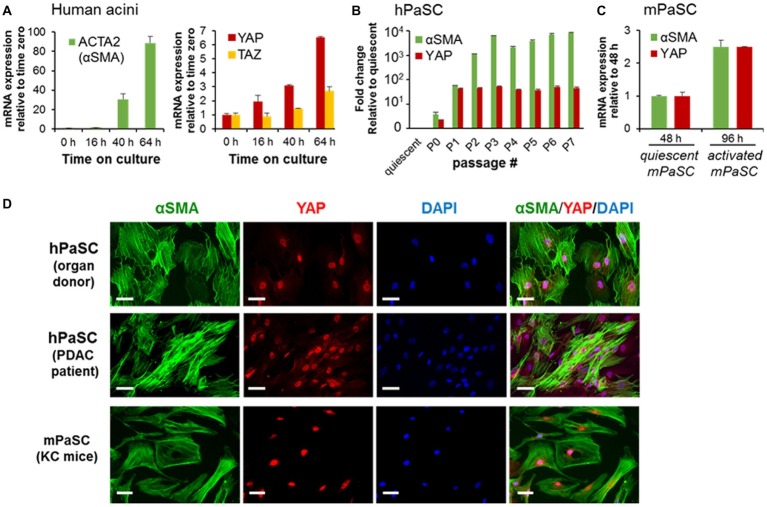
PaSC activation is associated with sustained YAP upregulation. **(A)** Activated PaSC were obtained in culture from an acinar-ductal-metaplasia (ADM) experimental model using pancreatic acini from cadaveric pancreata from human donors. As sown in the graphs, upregulation of YAP and its homolog TAZ in the ADM system was concomitant with upregulation of αSMA, a recognized maker of activated PaSC. αSMA and YAP mRNA expression were determined by qPCR. **(B)** Quiescent PaSC isolated from cadaveric human pancreata (hPaSC) did not express YAP, but YAP levels markedly increased during PaSC activation in culture in parallel with upregulation of αSMA. Similar results were obtained in PaSC obtained from WT mouse pancreas (mPaSC) **(C)**. Graphs in A-C show mean ± SEM; *n* = 3. **(D)** Immunofluorescence analysis of YAP (red) and αSMA (green) in hPDAC obtained from cadaveric human pancreata (organ donor) or pancreatic adenocarcinoma tumors (PDAC patient) and in mPaSC obtained from KC mice; nuclei were stained with DAPI (blue). Primary cells were expanded in culture using DMEM/F12 medium containing 10% FBS until 70–90% confluency and then immunostained with the indicated targets. Under these culture conditions, both hPaSC and mPaSC display YAP nuclear staining. Similar results were obtained in two additional independent experiments; Scale bar, 50 μM.

To further explore the role of YAP in PaSC biology, we measured levels and cellular location of YAP in quiescent and activated cultured PaSC obtained from human (hPaSC) and mouse (mPaSC) tissues. We first used a model of acinar-ductal-metaplasia (ADM) generated by culturing pancreatic exocrine cells from human cadaveric donor pancreata without pancreas pathology (Lugea et al., 2017). In this model that mimics parenchymal remodeling during CP initiation and progression, acinar cells rapidly die or dedifferentiate into ductal cells (not shown) and quiescent PaSC become activated and expand. In this system, PaSC exhibited marked upregulation of αSMA and concomitant upregulation of YAP and its homolog TAZ (Figure 2A).

We further found that YAP expression is linked to the activated state of PaSC. As shown in Figures 2B,C, quiescent, freshly isolated hPaSC, and mPaSC did not express YAP, but YAP levels markedly increased during cell activation in culture and remained elevated during passages. Moreover, as we observed in pancreas tissues, activated PaSC displayed robust YAP nuclear staining in culture conditions, likely associated with YAP transcriptional activity. As illustrated in Figure 2D, αSMA-positive PaSC obtained from normal human organ donor pancreata, resected human PDAC tumors or KC mice, all exhibit nuclear YAP. This staining pattern was also found in culture-activated mPaSC isolated from wild-type mice (not shown). We also found that, unlike reports using cancer and other cell types (Zhao et al., 2007; Hao et al., 2017), the number of PaSC displaying nuclear YAP is not affected by cell density (60–100%; not shown), a property likely related to tension induced by the contractile αSMA filaments in PaSC (Calvo et al., 2013).

### Pancreatic Stellate Cells Quiescence Induced by Inhibition of p38 MAPK Signaling and the Bromodomain and Extra-Terminal Bromodomain Family Proteins Results in Yes-Associated Protein 1 Downregulation

**Figure 3:**
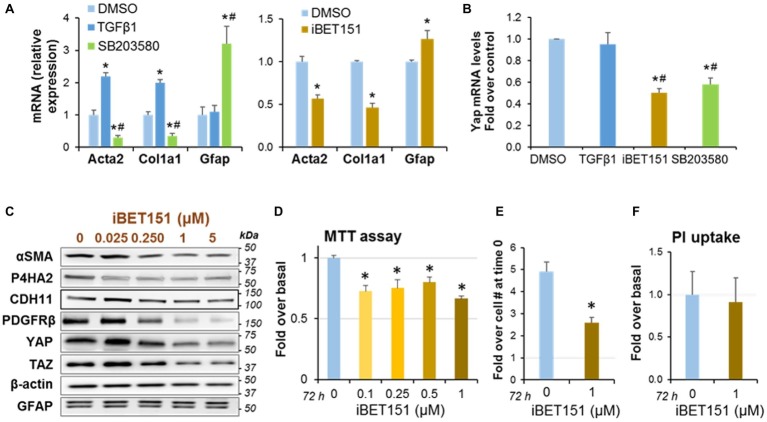
PaSC quiescence induced by a p38 MAP Kinase inhibitor or a BET family inhibitor is associated with YAP downregulation. Immortalized mouse PaSC (imPaSC) were treated with DMSO (control), the p38 MAPK inhibitor SB203580 (5 μM) or the BET inhibitor iBET151 (1 μM) for different times. In selected experiments, TGFβ1 (5 ng/ml) was used as positive control to induce upregulation of proteins found increased in activated PaSC (αSMA and Col1a1). **(A,B)** Graphs show mRNA levels of Acta2 (αSMA), Col1a1, GFAP (a marker of quiescent PaSC) and YAP measured by qPCR in imPaSC treated for 24 h with the indicated agents. Data is representative of three independent experiments. ^*^p < 0.05 vs. DMSO; ^#^*p* < 0.05 vs. TGFβ1 treatment. **(C)** Protein extracts from imPaSC treated for 24 h with different concentrations of iBET151 were analyzed to determine levels of YAP and TAZ and the indicated PaSC markers; representative immunoblots are shown. β-actin was used as loading control. As shown, BET inhibition decreases protein levels of several markers of PaSC activation but does not affect levels of the quiescent marker GFAP. Data is representative of three independent experiments. **(D–F)** imPaSC were treated for 72 h with iBET151 at the indicated concentrations, and metabolic activity (MTT assay), cell number and cell necrosis (PI uptake) were assessed. *Additionally*, Supplementary Figure S2A *shows protein levels of caspase-3 as a marker of caspase-dependent apoptosis*. **(D)** Graph shows MTT assay data after 72 h treatment. (E) Graph shows number of imPaSC after 72 h treatment with or without 1 μM iBET151. The number of cells at 0 and 72 h were assessed by trypan blue exclusion using a hemocytometer; data are expressed as fold increase over the cell number at time 0 (1.25 × 10^4^). **(E)** PI uptake was measured as indicated in “Materials and Methods” section. Graphs show ^*^p < 0.05 vs. no-iBET151 control. Data in graphs **(D–F)** represent the means ± SD; *n* = 3.

We next examined the effects of pharmacologic treatments that induce reversal of PaSC activation state into a quiescent state. This reversion was characterized by downregulation of the activation markers αSMA (encoded by the Acta2 gene) and collagen type I alpha 1 (encoded by the Col1a1 gene), upregulation of the quiescence marker glial fibrillary acidic protein (GFAP) and decrease in cell proliferation (Omary et al., 2007). We found that treatment with the p38 MAPK inhibitor SB203580 (Masamune et al., 2003) and with iBET151, a pan-specific inhibitor of the epigenetic transcriptional regulators Bromodomain and Extraterminal domain (BET) proteins (Kumar et al., 2017), induced quiescence in mPaSC (Figure 3A). By contrast, treatment with the known PaSC activator TGFβ1 resulted in marked upregulation of αSMA and Col1a1 in mPaSC (Figure 3A). Interestingly, both pharmacological inhibitors also significantly reduced YAP expression levels (62% reduction with 1 μM iBET151, and 48% with 0.5 μM SB203580; Figure 3B). Moreover, iBET151 reduced in a dose-dependent manner protein levels of the YAP homolog TAZ and the PaSC markers αSMA, CDH11, PDGFRβ and P4HA2, a collagen synthesizing enzyme (Figures 3B,C).

Compared to controls, iBET151 treatment also reduced PaSC metabolic activity, as assessed by MTT assay (Figure 3D), and cell numbers (Figure 3E). Differences in MTT absorbance values may reflect changes in cell proliferation and/or cell viability when metabolic events lead to necrosis or apoptosis. To determine whether iBET151 induces cell death, we assessed necrosis by measuring propidium iodide (PI) uptake and apoptosis by measuring protein levels of total and cleaved caspase-3. Our data indicate that iBET151 treatment did not change necrosis (Figure 3F) or apoptosis (Supplementary Figure S2A), suggesting that the BET inhibitor reduces cell proliferation, an effect consistent with a quiescent state.

### Yes-Associated Protein 1 Knockdown *Via* siRNA Transfection Reduces Cell Growth in Pancreatic Stellate Cells and Alters Expression of Genes Involved in Matrix Remodeling

**Figure 4:**
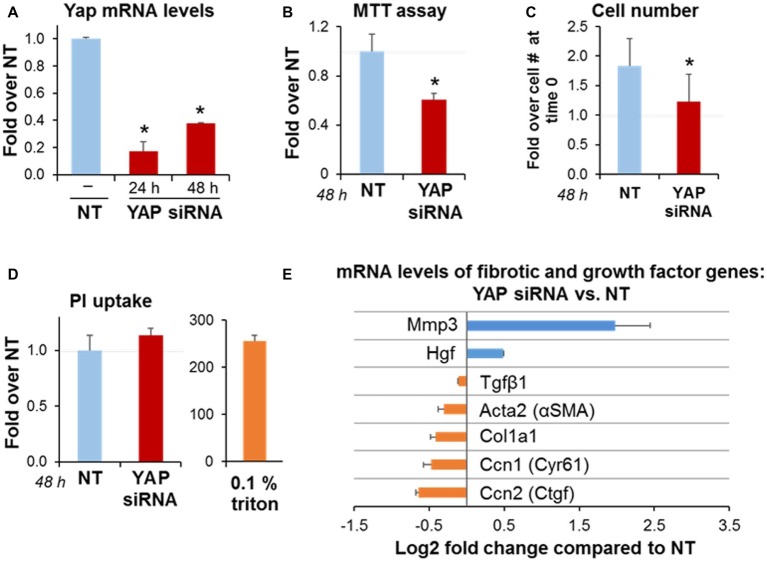
YAP siRNA decreases PaSC expansion in culture and alters expression of genes involved in matrix remodeling. imPaSC were transfected with nontargeting negative control (**NT**, 10 nmol/L) or a target siRNA (**YAP**, 10 nmol/L) mixed with lipofectamine RNAiMAX, and 24 h later cells were used for experiments. **(A)** Graph shows quantitation of YAP mRNA expression relative to negative control at 48 h post-transfection. Data are mean ± SD, *n* = 3; ^*^p < 0.05 vs. NT. **(B)** MTT assay for YAP siRNA-treated cells relative to NT. **(C)** The graph shows imPaSC numbers counted 0 and 48 h after treatment with either NT or YAP siRNA. The cell numbers were assessed by trypan blue exclusion using a hemocytometer, and expressed as a fold increase over time 0 (1.5 × 10^5^). **(D)** PI uptake was measured in cells treated with siRNA to detect any disrupted plasma membrane integrity. After incubation, a portion of cells was permeabilized for 5 min with 0.1% Triton X100 to establish a positive control. Data in graphs **(B–D)** are mean ± SD, *n* = 3–6 independent experiments; ^*^p < 0.05 vs. NT. Supplementary Figure S2B *shows protein levels of caspase-3 as a marker of caspase-dependent apoptosis*. **(E)** Graph shows mRNA levels of the indicated genes in YAP siRNA treated cells compared to NT. Data is mean ± SD, *n* = 5.

To gain insights into the role of YAP on activated PaSC, we transiently knocked down YAP mRNA in immortalized mPaSC *via* siRNA transfection as indicated in the Materials and Methods section. siRNA treatment resulted in 82% reduction in YAP mRNA levels at 24 h and 49% reduction at 48 h post-transfection (Figure 4A). At 48 h, we found in YAP siRNA-treated cells a 50% reduction in MTT absorbance values (MTT assay; Figure 4B), and a marked decrease in cell counts (Figure 4C). We did not observe morphological evidence of cell death in YAP siRNA-treated cells (not shown), necrosis (Figure 4D) or caspase-3-dependent apoptosis (Supplementary Figure S2B). Taken together, these data suggest that YAP regulates cell proliferation in activated PaSC.

As illustrated in Figure 4E, YAP siRNA knockdown in PaSC reduced a subset of genes including the previously identified YAP gene targets connective tissue growth factor (Ctgf) and cysteine rich angiogenic inducer 61 (Cyr61) that are involved in cell adhesion and ECM remodeling; the PaSC markers αSMA and Col1a1 that participate in PaSC morphology, matrix tension and collagen deposition, and TGFβ1, that stimulates fibroinflammatory responses in these cells. Interestingly, YAP siRNA treatment markedly increased levels of metalloproteinase 3 (MMP3), a protease involved in degradation of collagens and other ECM proteins during tissue remodeling. These findings support the concept that YAP represses MMP3 transcription in PaSC while promoting the expression of genes favoring collagen deposition and fibrosis. YAP knockdown also led to moderately increased expression of hepatocyte growth factor (HGF; Figure 4C), a growth factor that is secreted by PaSC and acts on epithelial and cancer cells regulating angiogenesis, tissue regeneration and cancer cell growth (Pothula et al., 2017). Overall, our findings indicate that YAP controls a variety of transcriptional gene networks in PaSC.

### PDGF Activates the Hippo Pathway and Increases Yes-Associated Protein 1 Phosphorylation in Pancreatic Stellate Cells

Rapid upregulation of PDGFRβ is a major event in the activation of PaSC from the quiescent phenotype, and PDGF binding to this receptor acts as a potent mitogen and induces migration in PaSC (Kordes et al., 2005). PDGF is secreted by different cell types including platelets, endothelial cells, and macrophages. In PDAC tissues, cancer cells and macrophages are likely a main source of PDGF and TGFβ1 with distinct effects on PaSC proliferation, migration and production of ECM and cytokines (Bachem et al., 2005; Omary et al., 2007; Apte et al., 2013; Biffi et al., 2019). PDGF induces cell growth and proliferation, in part by activating MAPK and PI3K/AKT signaling pathways (Heldin, 2013).

**Figure 5:**
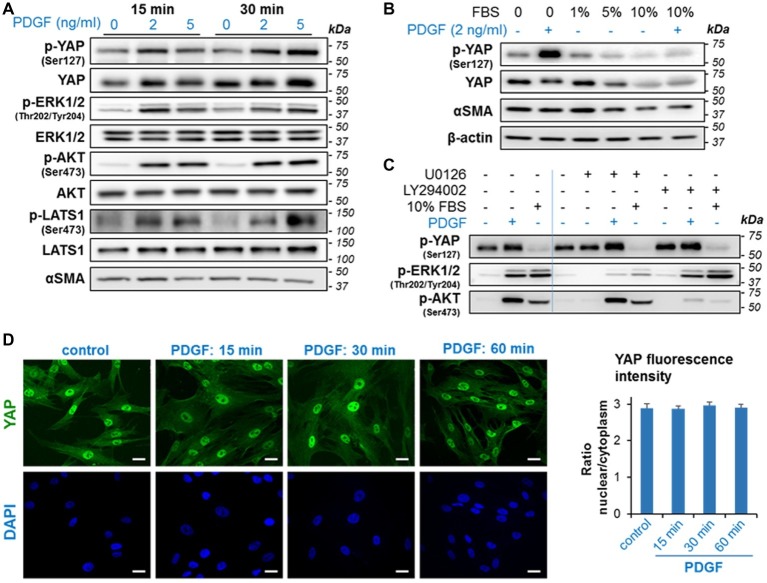
PDGF induces rapid phosphorylation of YAP and activation of growth signaling in imPaSC. **(A)** imPaSC cultured in DMEM containing 10% FBS, were serum-starved for 12 h and then treated with PDGF (2 and 5 ng/ml) for 15- and 30-min. Levels of total and phosphorylated forms of the indicated targets were measured by western blotting. As shown in the immunoblots, PDGF induced rapid activation of ERK1/2, AKT, and Hippo kinases (LATS1) and significantly increased levels of phosphorylated YAP (at Ser127). **(B)** Cells cultured in DMEM containing 10% FBS were serum-starved for 12 h and then cultured in media containing different concentrations of FBS. Twenty-four hours later, cells were treated for 30 min with and without 2 ng/ml PDGF. As shown in the immunoblots, 5 and 10% FBS reduced levels of total and phosphorylated YAP while PDGF markedly induced YAP phosphorylation. **(C)** imPaSC were serum-starved for 12 h, pre-treated with the MEK inhibitor U0126 (10 μM) or the PI3K/AKT inhibitor LY294002 (20 μM) for 1 h and then treated with or without 2 ng/ml PGDF or 10% FBS for 30 min. Levels of the indicated targets were measured by Western blotting. **(D)** Cells treated with 2 ng/ml PDGF for up to 60 min were IF stained using a YAP specific antibody; Scale bar, 50 μm. Quantified nuclear/cytoplasmic YAP fluorescence intensity is shown in **(E)**. Fluorescence was measured as indicated in *Materials and Methods* and expressed as nuclear/cytoplasmic ratio (mean ± SEM, *n* = 70–90 cells). As shown in the pictures, YAP staining was preferentially located in the nucleus in both control and PDGF-treated cells, and PDGF treatment did not alter YAP nuclear/cytoplasm ratios. Data in panels **A–E** are representative of three independent experiments.

Here, we investigated potential crosstalk between PDGF and YAP pathways in regulating PaSC growth in culture. Serum-starved imPaSC were treated with 2 or 5 ng/ml PDGF for different times. As expected, we found that PDGF induced rapid (within minutes) activation of ERK1/2 and AKT signaling in imPaSC, as indicated by increased phosphorylation of ERK1/2 at Thr202/Tyr204 and AKT at Ser473 (Figure 5A). Similar effects were found in primary human and mouse PaSC (not shown). PDGF treatment also led to rapid phosphorylation of YAP at Ser127, a site previously reported to be phosphorylated by the Hippo pathway kinase large tumor suppressor kinase 1 (LATS1) and to promote cytoplasmic retention and inactivation of YAP in several cell types (Zhao et al., 2007). In agreement, we found that PDGF induced LATS1 activation in imPaSC (Figure 5A).

Serum is a potent growth factor in activated PaSC and was reported to decrease YAP Ser127 phosphorylation and promote YAP nuclear translocation in HEK293A (Yu et al., 2012). To compare the effects of serum and PDGF on YAP activation, we cultured serum-starved imPaSC for 24 h in media containing different concentrations of FBS, and then treated the cells for 30 min with or without 2 ng/ml PDGF. As shown in Figure 5B, levels of p-YAP Ser127 decreased in cells treated with 5 and 10% FBS while PDGF increased YAP phosphorylation in both serum-starved and 10% FBS treated imPaSC (Figure 5B). To determine whether kinases downstream of ERK1/2 and AKT pathways mediate serum and PDGF effects on YAP phosphorylation at Ser127, we pre-treated imPaSC with the MEK inhibitor U0126 (10 μM) or the PI3K/AKT inhibitor LY294002 (20 μM) for 1 h, and then treated the cells with or without PGDF or 10% FBS for 30 min. As shown in Figure 5C, U0126 and LY294002 inhibited ERK1/2 and AKT activation, respectively in both PDGF- and serum treated imPaSC but did not alter levels of p-YAP. These data indicate that, at least at the time-point measured, YAP Ser127 phosphorylation elicited by PDGF is independent of ERK and AKT activation and is likely regulated by LATS1.

We next investigated whether PDGF-induced YAP Ser127 phosphorylation was associated with changes in YAP cellular localization or translocation of YAP from the nucleus to the cytoplasm. Surprisingly, we found that PDGF-induced YAP phosphorylation did not change YAP nuclear location, as determined by immunofluorescence staining (Figure 5D) and quantification of nuclear-to-cytoplasm YAP fluorescence intensity ratios (Figure 5E).

### Yes-Associated Protein 1 siRNA Knockdown Reduces PDGF-Induced Proliferation and Blunts the Persistent Activation of AKT and ERK Induced by Long-Term Stimulation With PDGF

**Figure 6:**
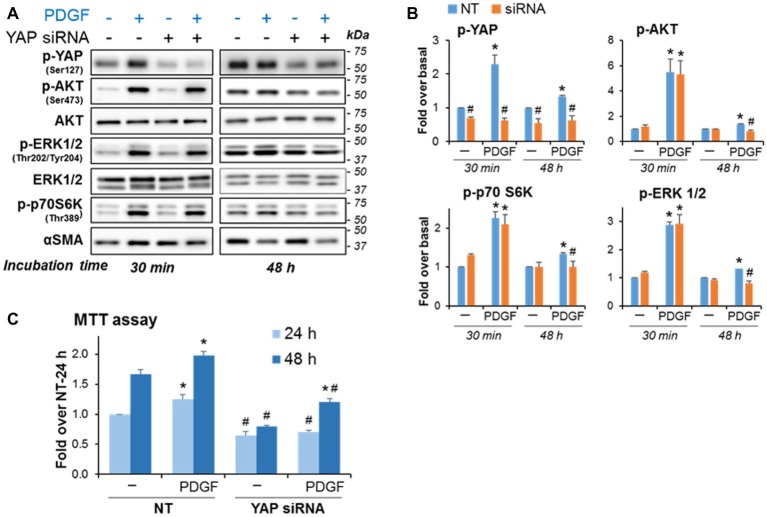
YAP knockdown blunts the persistent activation of AKT and ERK induced by long-term stimulation with PDGF and reduces PDGF-induced proliferation in imPaSC. Mock and YAP-transfected cells were treated with 2 ng/ml PDGF for the indicated times. **(A)** Levels of phosphorylated and total AKT, ERK1/2 and p70S6K (a kinase in the mTOR pathway) were assessed by Western blotting. αSMA was tested as a marker of activation. **(B)** Graphs show optical density (OD) of the immunoblots depicted in panel **A** determined by densitometry. As indicated in the graphs, YAP knockdown did not affect PDGF-induced early activation of AKT, ERK1/2 and mTOR signaling but reduced the long-term (48 h) PDGF effects on growth signaling. **(C)** YAP siRNA treatment also reduced basal and PDGF-induced cell proliferation as indicated by MTT assay. Data in graphs are mean ± SEM; *n* = 3. ^*^p < 0.05 vs. control (−); ^#^*p* < 0.05 vs. NT.

The data illustrated in Figure 4 suggest that YAP downregulation diminishes PaSC proliferation. To examine the potential involvement of YAP in PDGF-induced PaSC proliferation, we measured ERK1/2 and AKT activation state and PaSC proliferation in YAP siRNA- vs. mock-transfected cells treated with PDGF for up to 48 h. We found that in mock-transfected cells (Figure 6A), PDGF induced YAP Ser127 phosphorylation and phosphorylation-dependent activation of ERK1/2, AKT and p70S6K, an effector protein kinase in the mTORC1 pathway that we found regulates cell growth in PaSC (Yang et al., 2016). These PDGF effects persisted for at least 48 h. Compared to mock-transfected cells, YAP siRNA treatment did not affect early activation of AKT and ERK (at 30 min) but blunted long-term effects (at 48 h) of PDGF (Figures 6A,B). Compared to mock-transfected cells, YAP siRNA knockdown also diminished PDGF-induced proliferation (Figure 6C) but did not completely block PDGF effects, supporting YAP-dependent and -independent pathways regulating cell growth in imPaSC.

### Crosstalk Between Yes-Associated Protein 1 and Transforming Growth Factor Beta 1 Signaling in Cultured Pancreatic Stellate Cells

TGFβ1 is a potent activator of fibroinflammatory responses in PaSC (Apte et al., 1999; Xue et al., 2015). TGFβ1 effects on gene transcription are mediated *via* SMAD transcription factor signaling, and mounting evidence supports the existence of crosstalk between the Hippo pathway and SMAD signaling (Chen et al., 2019). For example, YAP and TAZ bind to SMAD2/3 within transcriptional complexes and regulate gene expression (Piersma et al., 2015), and YAP was reported to affect TGFβ1 signaling by upregulating the SMAD2/3 inhibitor SMAD7 (Qin et al., 2018).

**Figure 7:**
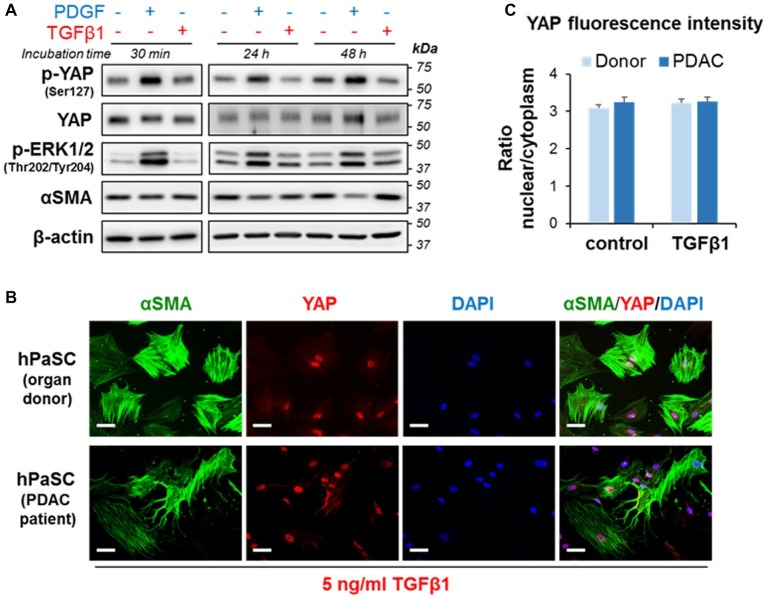
TGFβ1 does not affect does not affect YAP phosphorylation at Ser127 or YAP cellular localization in PaSC. **(A)** imPaSC were cultured in DMEM containing 10% FBS, starved in serum-free media for 12 h before experiment and then left untreated or treated with PDGF (2 ng/ml) or TGFβ1 (5 ng/ml). Immunoblots show protein levels of the indicated targets; β-actin was used as loading control. As shown in the pictures, PDGF induced sustained YAP phosphorylation at Ser127, whereas TGFβ1 had no effect. Similar results were found in primary PaSC obtained from mouse or human tissues (not shown). **(B)** hPaSC isolated from cadaveric pancreata (organ donors) or pancreatic tumor surgical resections (PDAC patients; see Figure 2D) were expanded in culture using DMEM/F12 medium containing 10% FBS until 70–90% confluency (as in Figure 2D) and then treated with 5 ng/ml TGFβ1 for 60 min. As observed in untreated cells (Figure 2D), YAP immunofluorescence was also mainly restricted to the nucleus in TGFβ1-treated cells. Data are representative of three independent experiments. Scale bar, 50 μm. **(C)** Graph shows relative YAP localization in untreated and TGFβ-treated cells obtained from donors or PDAC patients. Cellular YAP fluorescence is expressed as the nuclear/cytoplasmic ratio (mean ± SEM, *n* = 70–90 cells). As shown in the graph, TGFβ1 treatment had little effect on cellular localization of YAP.

Here, we first evaluate whether TGFβ1 modulates YAP levels and phosphorylation state in activated PaSC. We found that, unlike PDGF (Figures 5, 6), TGFβ1 (5 ng/ml) did not alter YAP protein levels or phosphorylation at Ser127 at any of the time-points examined (30 min, 24 h, and 48 h) (Figure 7A). Like PDGF, TGFβ1 stimulation had no perceptible effect on YAP nuclear localization. In this respect, we found that 100% of αSMA-positive hPaSC exhibit nuclear YAP both before (Figure 2D) and after 60 min treatment with TGFβ1 (Figure 7B) and that TGFβ1 treatment did not modify the nuclear-to-cytoplasmic YAP immunofluorescence ratios observed in non-treated hPaSC (Figure 7C). These findings suggest that under our culture conditions, YAP cellular localization in activated PaSC is not controlled by YAP Ser127 phosphorylation.

**Figure 8:**
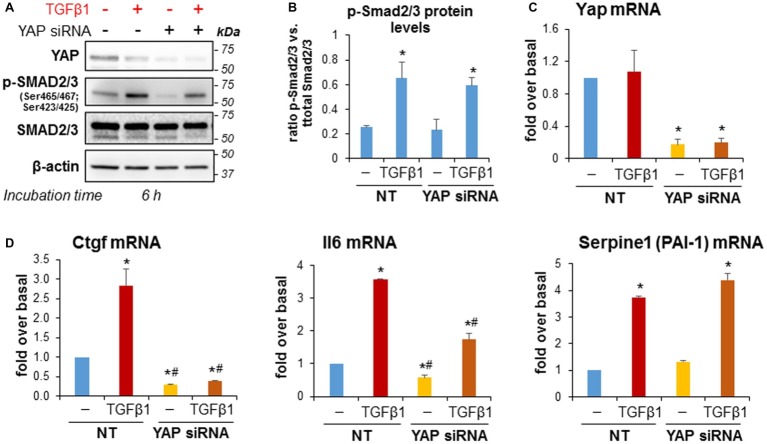
YAP siRNA significantly reduced TGFβ1-induced upregulation of Ctgf and IL-6 in imPaSC. **(A)** Twenty-four hours after transfection, mock and YAP-transfected imPaSC were treated with 5 ng/ml TGFβ1 for 6 h. As shown in the immunoblots, TGFβ1-induced activation of the SMAD2/3 pathway was similar in mock and YAP siRNA-treated cells. Similar results were obtained in three independent experiments. **(B)** Graph shows optical density (OD) of the immunoblots depicted in panel **A** determined by densitometry. **(C,D)** Twenty-four hours after transfection, mock (NT) and YAP-transfected imPaSC were treated with 5 ng/ml TGFβ1 for 6 h. mRNA levels of Yap **(C)**, Ccn1 (Ctgf), Il6, and the TGFβ1 target gene Serpine 1 (PAI-1) **(D)** were measured by qPCR and quantified relative to those of 18S rRNA. Data in graphs in panels **B–D** represent means ± SEM from three to five independent experiments; ^*^p < 0.05 vs. NT-not treated (−); ^#^*p* < 0.05 vs. NT-TGFβ1.

We next studied whether siRNA-mediated YAP knockdown would alter TGFβ1-induced SMAD2/3 activation and fibrosing responses in imPaSC. After 24 h of transfection, imPaSC were treated with TGFβ1 (5 ng/ml) for up to 6 h and levels of phosphorylated or total SMAD2/3 proteins were measured in cell lysates by Western blotting. We found that TGFβ1 induces rapid SMAD2/3 phosphorylation in non-transfected cells that persists for at least 6 h (not shown). As expected, YAP knockdown (Figure 8C) did not affect TGFβ1-induced Smad2/3 phosphorylation in imPaSC (Figures 8A,B). However, YAP knockdown almost completely abolished TGFβ1-induced Ctgf, and significantly diminished interleukin 6 (IL6) expression in these cells (Figure 8D). Interestingly, YAP knockdown did not affect the marked upregulation of plasminogen activator inhibitor 1 (PAI-1) elicited by TGFβ1 (Figure 8D), implying that YAP and TGFβ1 cooperate in the transcription of distinct gene networks in PaSC.

## Discussion

CP and PDAC are devastating pancreatic diseases characterized by high morbidity and mortality and limited treatment options (Garrido-Laguna and Hidalgo, 2015; Adamska et al., 2017; Kleeff et al., 2017). It is widely accepted that activated, myofibroblast-like PaSC modulate disease states and are major contributors to the extensive pancreatic desmoplasia and fibrosis observed in CP and PDAC (Apte et al., 2013). However, the mechanisms that govern PaSC activation, proliferation and fibroinflammatory responses during disease initiation and progression are not completely understood. Moreover, the contribution of key cellular pathways such as the Hippo/YAP pathway, that is involved in CP and PDAC progression (Murakami et al., 2017; Zhao et al., 2017), to the pathobiology of PaSC is poorly defined. This area of research is important because therapies directed to those specific pathways may have an impact on PaSC responses and PaSC modulation of the disease.

A previous study from this laboratory showed immunohistochemical evidence of YAP expression within activated, αSMA-positive PaSC in human CP and PDAC tumors, and KC mice (Morvaridi et al., 2015). In this study, we found that YAP expression upregulates in the diseased pancreas in parallel with PaSC activation. Moreover, YAP expression in cultured PaSC is linked to the cell activation state. Activated PaSC obtained either from normal mouse or human pancreas, or from human or mouse PDAC tumor tissues exhibit preferential YAP nuclear staining, suggesting that YAP is transcriptionally active in these cells. Preferential YAP nuclear localization was a constant feature in cultured PaSC regardless of cell confluency (60–100%). These findings are consistent with previous studies demonstrating that YAP cellular localization is regulated by cell morphology and the F-actin cytoskeleton (Wada et al., 2011; Calvo et al., 2013). Cell spreading, a feature of myofibroblasts including PaSC, leads to YAP activation and translocation to the nucleus (Wada et al., 2011). Moreover, it was reported that incorporation of αSMA into stress fibers in mesenchymal cells promotes YAP nuclear translocation and YAP transcriptional activity (Talele et al., 2015).

Recent studies observed that treatment with BET inhibitors effectively reduced fibrosis and PDAC progression in mice, and implied that the activated state of PaSC was attenuated (Ding et al., 2015; Mazur et al., 2015). BRD4, a major BET protein, has also been implicated as a modulator of YAP/TAZ transcriptional activity at distinct promoters in cancer cells (Zanconato et al., 2018). Consistent with these reports, we found that treatment with the pan-specific BET protein inhibitor iBET151 reduced both mRNA and protein levels of YAP, and induced quiescence in PaSC, extending the mechanisms by which BET proteins regulate YAP/TAZ activities to the level of YAP/TAZ expression. Like BET inhibitors, we found that the p38 MAPK inhibitor SB 203580 also induced quiescence and reduced YAP expression in PaSC, further supporting a close association between YAP levels and PaSC activation state. Treatment with the mTOR inhibitor, KU63794, that decreased proliferation and ECM production in PaSC (Yang et al., 2016), also reduced YAP levels by 70% (data not shown). These findings support a key role of YAP in the activated PaSC phenotype. Further studies are needed to elucidate the mechanisms whereby p38 MAPK and mTOR pathways may intersect with BET proteins and other regulators of YAP/TAZ expression and activity.

Growth factors such as PDGF, IGF1, and TGFβ1 were identified as inducers of PaSC activation into proliferative myofibroblasts (Apte et al., 1999, 2013; Luttenberger et al., 2000; Schneider et al., 2001; Mews et al., 2002; Yang et al., 2016). This study addresses crosstalk between PDGF or TGFβ1 signaling and YAP in PaSC. PaSC activation is associated with rapid and marked upregulation of PDGFRβ, and PDGF is a potent mitogen in PaSC. As expected, we found that PDGF induced sustained activation of AKT and ERK signaling. We also found that PDGF enhanced LATS Ser473 and YAP Ser127 phosphorylation. In other cell types, negative regulation by LATS protein kinases that phosphorylate YAP Ser127 typically restrict nuclear translocation and decrease co-transcriptional activity. Here, PDGF treatment dramatically enhanced YAP Ser127 phosphorylation but had no perceptible effect on preferential YAP nuclear localization in PaSC. These findings are consistent with a previous report demonstrating that pharmacological inhibition of PDGFRβ or PDGFRβ siRNA silencing decreased levels of YAP Ser127 and induced YAP translocation to the cytoplasm in cholangiocarcinoma cells (Smoot et al., 2018), suggesting a distinct role for PDGF signaling in regulating YAP activation. In this study, we found that YAP knockdown blunted the PDGF-induced persistent activation of AKT and ERK, and reduced PDGF-induced proliferation, further supporting a crosstalk between PDGF and YAP signaling.

TGFβ1 treatment on the other hand had no effect on the levels of total or Ser127-phosphorylated YAP. Further, whereas TGFβ1-induced Smad2/3 pathway activation was unaffected, and TGFβ1-induced of CTGF and IL-6 upregulation were markedly blocked by YAP knockdown in PaSC. Thus, YAP modulates gene transcription elicited by TGFβ1/SMAD pathways in PaSC, as previously shown in other cell types (Zheng and Pan, 2019). These findings support YAP activity in PaSC as an important regulator of TGFβ1-induced fibroinflammatory responses in CP and PDAC.

Taken together, our data further substantiate the importance of nuclear YAP previously detected in PaSC in CP and PDAC stroma (Morvaridi et al., 2015). In particular, our novel findings clarify a key role for YAP in regulating PaSC activation state, proliferation, and fibroinflammatory responses conducive to CP and PDAC progression. These results highlight YAP signaling as an important therapeutic target to prevent or halt the expansion of desmoplasia in pancreatic disorders. Further studies are necessary to explore the complex crosstalk between distinct signaling systems including YAP, TGFβ1, and mitogenic signals regulating the activation state of PaSC in these disorders.

## Data Availability Statement

The datasets generated for this study are available on request to the corresponding author.

## Ethics Statement

Animal studies were approved by the Institutional Animal Care and Use Committee at Cedars-Sinai Medical Center (Los Angeles, CA) and were in accordance with the NIH Guide for the Care and Use of Laboratory Animals. The use of human tissues was performed in accordance with regulations and IRB protocols approved by the Institutional Review Board at Cedars-Sinai Medical Center (Los Angeles, CA; IRB protocols Pro31101 and Pro32114).

## Author Contributions

CH, JY, H-YS, RW, MZ, and AL participated in the design and coordination of the study, performed experiments, data acquisition, analysis, and interpretations. CH, RW, SP, and AL participated in study concept and manuscript writing. LL and QX reviewed and approved the final manuscript. All authors read and approved the final manuscript.

### Conflict of Interest

The authors declare that the research was conducted in the absence of any commercial or financial relationships that could be construed as a potential conflict of interest.

## Supplementary Material

SUPPLEMENTARY FIGURE S1 **(A)** Pancreas damage in mice during recurrent acute pancreatitis (RAP). C57BL/6 mice were subjected to two episodes of acute cerulein pancreatitis (days 1 and 3; 1 day apart). Each episode consisted of 7 hourly intraperitoneal injections of 50 μg/kg cerulein or saline as vehicle control. Tissues were collected 2 days after the second episode of pancreatitis (day 5; d5). Panels show representative H&E-stained pancreatic tissue sections from mice treated with saline (control; panels **a,b**) or cerulein (RAP-d5; panels **c,d**); five to six mice per group. Dotted boxes in panels **a,****c** (scale bar, 200 μm) are shown at higher magnification (scale bar 50 μm) in panels **b,****d**, respectively. As shown in the images, pancreas from control mice (panels **a,b**) exhibit a normal exocrine parenchyma composed of groups of acinar cells (acini) enriched in digestive enzymes (zymogen granules). Islets (endocrine cells) are also observed in the pictures. At day 5 after RAP (panels **c,d**), pancreatic tissues display acinar cell necrosis, loss of normal parenchyma, and stromal expansion enriched in immune cells and PaSC. As shown in Figure 1C, α-SMA-positive PaSC were found in RAP-5d but not in control tissues. **(B)** Pancreas histology in wild type and Kras mice. Representative IHC images showing αSMA staining (brown) in pancreas tissues of 3 month-old wild-type and Ptf1-Cre; LSL-Kras^G12D/+^ (KC) mice. In wild-type mice, precancerous lesions (pancreatic intraepithelial neoplasia or PanIN) are not present and positive staining is found only in the wall of blood vessels (see arrow head). In KC mice, abundant αSMA-positive cells are found in the stroma surrounding PanINs, indicating activated PaSC.

SUPPLEMENTARY FIGURE S2 **(A)** Immortalized mouse PaSC (imPaSC) were treated with the BET inhibitor, iBET151 for 72 h. **(B)** imPaSC were transfected with non-targeting negative control (**NT**, 10 nmol/L) or a targeted siRNA (**YAP**, 10 nmol/L), and proteins extracted 48 h later. Immunoblots show levels of full length (~35 kDa) and cleaved (~17 kDa) Caspase-3. GAPDH was used as loading control. Compared to controls, neither iBET151 nor YAP siRNA treatments increased levels of cleaved Caspase-3. Data are representative of three to four independent experiments.

## References

[ref1] Adamska A. Domenichini A. Falasca M. (2017). Pancreatic ductal adenocarcinoma: current and evolving therapies. Int. J. Mol. Sci. 18:1338. 10.3390/ijms18071338 PMC5535831 28640192

[ref2] Ansari D. Ohlsson H. Althini C. Bauden M. Zhou Q. Hu D. (2019). The hippo signaling pathway in pancreatic cancer. Anticancer Res. 39, 3317–3321. 10.21873/anticanres.13474 31262852

[ref3] Apte M. V. Haber P. S. Darby S. J. Rodgers S. C. McCaughan G. W. Korsten M. A. (1999). Pancreatic stellate cells are activated by proinflammatory cytokines: implications for pancreatic fibrogenesis. Gut 44, 534–541. 10075961 10.1136/gut.44.4.534 PMC1727467

[ref4] Apte M. V. Pirola R. C. Wilson J. S. (2012). Pancreatic stellate cells: a starring role in normal and diseased pancreas. Front. Physiol. 3:344. 10.3389/fphys.2012.00344 22973234 PMC3428781

[ref5] Apte M. V. Wilson J. S. Lugea A. Pandol S. J. (2013). A starring role for stellate cells in the pancreatic cancer microenvironment. Gastroenterology 144, 1210–1219. 10.1053/j.gastro.2012.11.037 23622130 PMC3729446

[ref6] Bachem M. G. Schunemann M. Ramadani M. Siech M. Beger H. Buck A. (2005). Pancreatic carcinoma cells induce fibrosis by stimulating proliferation and matrix synthesis of stellate cells. Gastroenterology 128, 907–921. 10.1053/j.gastro.2004.12.036 15825074

[ref7] Biffi G. Oni T. E. Spielman B. Hao Y. Elyada E. Park Y. (2019). IL1-induced JAK/STAT signaling is antagonized by TGFbeta to shape CAF heterogeneity in pancreatic ductal adenocarcinoma. Cancer Discov. 9, 282–301. 10.1158/2159-8290.CD-18-0710 30366930 PMC6368881

[ref8] Calvo F. Ege N. Grande-Garcia A. Hooper S. Jenkins R. P. Chaudhry S. I. (2013). Mechanotransduction and YAP-dependent matrix remodelling is required for the generation and maintenance of cancer-associated fibroblasts. Nat. Cell Biol. 15, 637–646. 10.1038/ncb2756 23708000 PMC3836234

[ref9] Cebola I. Rodriguez-Segui S. A. Cho C. H. Bessa J. Rovira M. Luengo M. (2015). TEAD and YAP regulate the enhancer network of human embryonic pancreatic progenitors. Nat. Cell Biol. 17, 615–626. 10.1038/ncb3160 25915126 PMC4434585

[ref10] Chang H. H. Moro A. Takakura K. Su H. Y. Mo A. Nakanishi M. (2017). Incidence of pancreatic cancer is dramatically increased by a high fat, high calorie diet in KrasG12D mice. PLoS One 12:e0184455. 10.1371/journal.pone.0184455 28886117 PMC5590955

[ref11] Chen Y. A. Lu C. Y. Cheng T. Y. Pan S. H. Chen H. F. Chang N. S. (2019). WW domain-containing proteins YAP and TAZ in the hippo pathway as key regulators in stemness maintenance, tissue homeostasis, and tumorigenesis. Front. Oncol. 9:60. 10.3389/fonc.2019.00060 30805310 PMC6378284

[ref12] Dawson D. W. Hertzer K. Moro A. Donald G. Chang H. H. Go V. L. (2013). High-fat, high-calorie diet promotes early pancreatic neoplasia in the conditional KrasG12D mouse model. Cancer Prev. Res. 6, 1064–1073. 10.1158/1940-6207.CAPR-13-0065 PMC3835151 23943783

[ref13] Ding N. Hah N. Yu R. T. Sherman M. H. Benner C. Leblanc M. (2015). BRD4 is a novel therapeutic target for liver fibrosis. Proc. Natl. Acad. Sci. USA 112, 15713–15718. 10.1073/pnas.1522163112. 26644586 PMC4697417

[ref14] Dupont S. Morsut L. Aragona M. Enzo E. Giulitti S. Cordenonsi M. (2011). Role of YAP/TAZ in mechanotransduction. Nature 474, 179–183. 10.1038/nature10137 21654799

[ref15] Eibl G. Rozengurt E. (2019). KRAS, YAP, and obesity in pancreatic cancer: a signaling network with multiple loops. Semin. Cancer Biol. 54, 50–62. 10.1016/j.semcancer.2017.10.007 29079305 PMC5916582

[ref16] Erkan M. Adler G. Apte M. V. Bachem M. G. Buchholz M. Detlefsen S. (2012). StellaTUM: current consensus and discussion on pancreatic stellate cell research. Gut 61, 172–178. 10.1136/gutjnl-2011-301220 22115911 PMC3245897

[ref17] Garrido-Laguna I. Hidalgo M. (2015). Pancreatic cancer: from state-of-the-art treatments to promising novel therapies. Nat. Rev. Clin. Oncol. 12, 319–334. 10.1038/nrclinonc.2015.53. 25824606

[ref18] Gukovskaya A. S. Gorelick F. S. Groblewski G. E. Mareninova O. A. Lugea A. Antonucci L. (2019). Recent insights into the pathogenic mechanism of pancreatitis: role of acinar cell organelle disorders. Pancreas 48, 459–470. 10.1097/MPA.0000000000001298 30973461 PMC6461375

[ref19] Hao F. Xu Q. Zhao Y. Stevens J. V. Young S. H. Sinnett-Smith J. (2017). Insulin receptor and GPCR crosstalk stimulates YAP *via* PI3K and PKD in pancreatic cancer cells. Mol. Cancer Res. 15, 929–941. 10.1158/1541-7786.MCR-17-0023 28360038 PMC5645013

[ref20] Heldin C. H. (2013). Targeting the PDGF signaling pathway in tumor treatment. Cell Commun. Signal 11:97. 10.1186/1478-811X-11-97 24359404 PMC3878225

[ref21] Kapoor A. Yao W. Ying H. Hua S. Liewen A. Wang Q. (2014). Yap1 activation enables bypass of oncogenic Kras addiction in pancreatic cancer. Cell 158, 185–197. 10.1016/j.cell.2014.06.003. 24954535 PMC4109295

[ref22] Kirkegard J. Mortensen F. V. Cronin-Fenton D. (2017). Chronic pancreatitis and pancreatic cancer risk: a systematic review and meta-analysis. Am. J. Gastroenterol. 112, 1366–1372. 10.1038/ajg.2017.218 28762376

[ref23] Kleeff J. Whitcomb D. C. Shimosegawa T. Esposito I. Lerch M. M. Gress T. (2017). Chronic pancreatitis. Nat. Rev. Dis. Primers. 3:17060. 10.1038/nrdp.2017.60 28880010

[ref24] Kordes C. Brookmann S. Haussinger D. Klonowski-Stumpe H. (2005). Differential and synergistic effects of platelet-derived growth factor-BB and transforming growth factor-beta1 on activated pancreatic stellate cells. Pancreas 31, 156–167. 10.1097/01.mpa.0000168222.05591.a0 16025003

[ref25] Kumar K. DeCant B. T. Grippo P. J. Hwang R. F. Bentrem D. J. Ebine K. (2017). BET inhibitors block pancreatic stellate cell collagen I production and attenuate fibrosis in vivo. JCI Insight 2:e88032. 10.1172/jci.insight.88032 28194432 PMC5291732

[ref26] Lew D. Afghani E. Pandol S. (2017). Chronic pancreatitis: current status and challenges for prevention and treatment. Dig. Dis. Sci. 62, 1702–1712. 10.1007/s10620-017-4602-2 28501969 PMC5507364

[ref27] Lugea A. Nan L. French S. W. Bezerra J. A. Gukovskaya A. S. Pandol S. J. (2006). Pancreas recovery following cerulein-induced pancreatitis is impaired in plasminogen-deficient mice. Gastroenterology 131, 885–899. 10.1053/j.gastro.2006.06.023 16952557 PMC1636452

[ref28] Lugea A. Waldron R. T. Mareninova O. A. Shalbueva N. Deng N. Su H. Y. (2017). Human pancreatic acinar cells: proteomic characterization, physiologic responses, and organellar disorders in ex vivo pancreatitis. Am. J. Pathol. 187, 2726–2743. 10.1016/j.ajpath.2017.08.017 28935577 PMC5718097

[ref29] Luttenberger T. Schmid-Kotsas A. Menke A. Siech M. Beger H. Adler G. (2000). Platelet-derived growth factors stimulate proliferation and extracellular matrix synthesis of pancreatic stellate cells: implications in pathogenesis of pancreas fibrosis. Lab. Investig. 80, 47–55. 10.1038/labinvest.3780007 10653002

[ref30] Majumder S. Chari S. T. (2016). Chronic pancreatitis. Lancet 387, 1957–1966. 10.1016/S0140-6736(16)00097-0 26948434

[ref31] Mannaerts I. Leite S. B. Verhulst S. Claerhout S. Eysackers N. Thoen L. F. (2015). The hippo pathway effector YAP controls mouse hepatic stellate cell activation. J. Hepatol. 63, 679–688. 10.1016/j.jhep.2015.04.011 25908270

[ref32] Masamune A. Satoh M. Kikuta K. Sakai Y. Satoh A. Shimosegawa T. (2003). Inhibition of p38 mitogen-activated protein kinase blocks activation of rat pancreatic stellate cells. J. Pharmacol. Exp. Ther. 304, 8–14. 10.1124/jpet.102.040287 12490569

[ref33] Mazur P. K. Herner A. Mello S. S. Wirth M. Hausmann S. Sanchez-Rivera F. J. (2015). Combined inhibition of BET family proteins and histone deacetylases as a potential epigenetics-based therapy for pancreatic ductal adenocarcinoma. Nat. Med. 21, 1163–1171. 10.1038/nm.3952 26390243 PMC4959788

[ref34] Mews P. Phillips P. Fahmy R. Korsten M. Pirola R. Wilson J. (2002). Pancreatic stellate cells respond to inflammatory cytokines: potential role in chronic pancreatitis. Gut 50, 535–541. 10.1136/gut.50.4.535 11889076 PMC1773172

[ref35] Morvaridi S. Dhall D. Greene M. I. Pandol S. J. Wang Q. (2015). Role of YAP and TAZ in pancreatic ductal adenocarcinoma and in stellate cells associated with cancer and chronic pancreatitis. Sci. Rep. 5:16759. 10.1038/srep16759 26567630 PMC4645184

[ref36] Murakami S. Shahbazian D. Surana R. Zhang W. Chen H. Graham G. T. (2017). Yes-associated protein mediates immune reprogramming in pancreatic ductal adenocarcinoma. Oncogene 36, 1232–1244. 10.1038/onc.2016.288 27546622 PMC5322249

[ref37] Ohlund D. Handly-Santana A. Biffi G. Elyada E. Almeida A. S. Ponz-Sarvise M. (2017). Distinct populations of inflammatory fibroblasts and myofibroblasts in pancreatic cancer. J. Exp. Med. 214, 579–596. 10.1084/jem.20162024 28232471 PMC5339682

[ref38] Omary M. B. Lugea A. Lowe A. W. Pandol S. J. (2007). The pancreatic stellate cell: a star on the rise in pancreatic diseases. J. Clin. Invest. 117, 50–59. 10.1172/jci30082 17200706 PMC1716214

[ref39] Pandol S. Gukovskaya A. Edderkaoui M. Dawson D. Eibl G. Lugea A. (2012). Epidemiology, risk factors, and the promotion of pancreatic cancer: role of the stellate cell. J. Gastroenterol. Hepatol. 27(Suppl. 2), 127–134. 10.1111/j.1440-1746.2011.07013.x 22320930 PMC3736749

[ref40] Peery A. F. Dellon E. S. Lund J. Crockett S. D. McGowan C. E. Bulsiewicz W. J. (2012). Burden of gastrointestinal disease in the United States: 2012 update. Gastroenterology 143, 1179–1187.e3. 10.1053/j.gastro.2012.08.002 22885331 PMC3480553

[ref41] Piersma B. Bank R. A. Boersema M. (2015). Signaling in fibrosis: TGF-beta, WNT, and YAP/TAZ converge. Front. Med. 2:59. 10.3389/fmed.2015.00059 PMC4558529 26389119

[ref42] Pothula S. P. Xu Z. Goldstein D. Merrett N. Pirola R. C. Wilson J. S. (2017). Targeting the HGF/c-MET pathway: stromal remodelling in pancreatic cancer. Oncotarget 8, 76722–76739. 10.18632/oncotarget.20822 29100344 PMC5652738

[ref43] Qin Z. Xia W. Fisher G. J. Voorhees J. J. Quan T. (2018). YAP/TAZ regulates TGF-beta/Smad3 signaling by induction of Smad7 *via* AP-1 in human skin dermal fibroblasts. Cell Commun. Signal 16:18. 10.1186/s12964-018-0232-3 29695252 PMC5918965

[ref44] Row S. Liu Y. Alimperti S. Agarwal S. K. Andreadis S. T. (2016). Cadherin-11 is a novel regulator of extracellular matrix synthesis and tissue mechanics. J. Cell Sci. 129, 2950–2961. 10.1242/jcs.183772 27311482 PMC5004872

[ref45] Rozengurt E. Sinnett-Smith J. Eibl G. (2018). Yes-associated protein (YAP) in pancreatic cancer: at the epicenter of a targetable signaling network associated with patient survival. Signal Transduct. Target. Ther. 3:11. 10.1038/s41392-017-0005-2 29682330 PMC5908807

[ref46] Schneider E. Schmid-Kotsas A. Zhao J. Weidenbach H. Schmid R. M. Menke A. (2001). Identification of mediators stimulating proliferation and matrix synthesis of rat pancreatic stellate cells. Am. J. Physiol. Cell Physiol. 281, C532–C543. 10.1152/ajpcell.2001.281.2.C532 11443052

[ref47] Smoot R. L. Werneburg N. W. Sugihara T. Hernandez M. C. Yang L. Mehner C. (2018). Platelet-derived growth factor regulates YAP transcriptional activity *via* Src family kinase dependent tyrosine phosphorylation. J. Cell. Biochem. 119, 824–836. 10.1002/jcb.26246 28661054 PMC5705491

[ref48] Song X. Qian X. Shen M. Jiang R. Wagner M. B. Ding G. (2015). Protein kinase C promotes cardiac fibrosis and heart failure by modulating galectin-3 expression. Biochim. Biophys. Acta 1853, 513–521. 10.1016/j.bbamcr.2014.12.001 25489662

[ref49] Su H. Y. Waldron R. T. Gong R. Ramanujan V. K. Pandol S. J. Lugea A. (2016). The unfolded protein response plays a predominant homeostatic role in response to mitochondrial stress in pancreatic stellate cells. PLoS One 11:e0148999. 10.1371/journal.pone.0148999 26849807 PMC4743835

[ref50] Talele N. P. Fradette J. Davies J. E. Kapus A. Hinz B. (2015). Expression of alpha-smooth muscle actin determines the fate of mesenchymal stromal cells. Stem Cell Rep. 4, 1016–1030. 10.1016/j.stemcr.2015.05.004 PMC4471834 26028530

[ref51] Wada K. Itoga K. Okano T. Yonemura S. Sasaki H. (2011). Hippo pathway regulation by cell morphology and stress fibers. Development 138, 3907–3914. 10.1242/dev.070987 21831922

[ref52] Xue J. Sharma V. Hsieh M. H. Chawla A. Murali R. Pandol S. J. (2015). Alternatively activated macrophages promote pancreatic fibrosis in chronic pancreatitis. Nat. Commun. 6:7158. 10.1038/ncomms8158 25981357 PMC4632846

[ref53] Yang D. Forsmark C. E. (2017). Chronic pancreatitis. Curr. Opin. Gastroenterol. 33, 396–403. 10.1097/MOG.0000000000000377 28771447

[ref54] Yang J. Waldron R. T. Su H. Y. Moro A. Chang H. H. Eibl G. (2016). Insulin promotes proliferation and fibrosing responses in activated pancreatic stellate cells. Am. J. Physiol. Gastrointest. Liver Physiol. 311, G675–G687. 10.1152/ajpgi.00251.2016 27609771 PMC5142202

[ref55] Yu F. X. Zhao B. Panupinthu N. Jewell J. L. Lian I. Wang L. H. (2012). Regulation of the hippo-YAP pathway by G-protein-coupled receptor signaling. Cell 150, 780–791. 10.1016/j.cell.2012.06.037 22863277 PMC3433174

[ref56] Zanconato F. Battilana G. Forcato M. Filippi L. Azzolin L. Manfrin A. (2018). Transcriptional addiction in cancer cells is mediated by YAP/TAZ through BRD4. Nat. Med. 24, 1599–1610. 10.1038/s41591-018-0158-8 30224758 PMC6181206

[ref57] Zhang W. Nandakumar N. Shi Y. Manzano M. Smith A. Graham G. (2014). Downstream of mutant KRAS, the transcription regulator YAP is essential for neoplastic progression to pancreatic ductal adenocarcinoma. Sci. Signal. 7:ra42. 10.1126/scisignal.2005049 24803537 PMC4175524

[ref58] Zhao X. Wang X. Fang L. Lan C. Zheng X. Wang Y. (2017). A combinatorial strategy using YAP and pan-RAF inhibitors for treating KRAS-mutant pancreatic cancer. Cancer Lett. 402, 61–70. 10.1016/j.canlet.2017.05.015 28576749

[ref59] Zhao B. Wei X. Li W. Udan R. S. Yang Q. Kim J. (2007). Inactivation of YAP oncoprotein by the hippo pathway is involved in cell contact inhibition and tissue growth control. Genes Dev. 21, 2747–2761. 10.1101/gad.1602907 17974916 PMC2045129

[ref60] Zheng Y. Pan D. (2019). The hippo signaling pathway in development and disease. Dev. Cell 50, 264–282. 10.1016/j.devcel.2019.06.003 31386861 PMC6748048

