# Supplementary figures and images for: Yes-Associated Protein 1 Plays Major Roles in Pancreatic Stellate Cell Activation and Fibroinflammatory Responses
Source: Front Physiol. 2019 Dec 3;10:1467. doi: 10.3389/fphys.2019.01467 (PMC6901825; doi:10.3389/fphys.2019.01467)

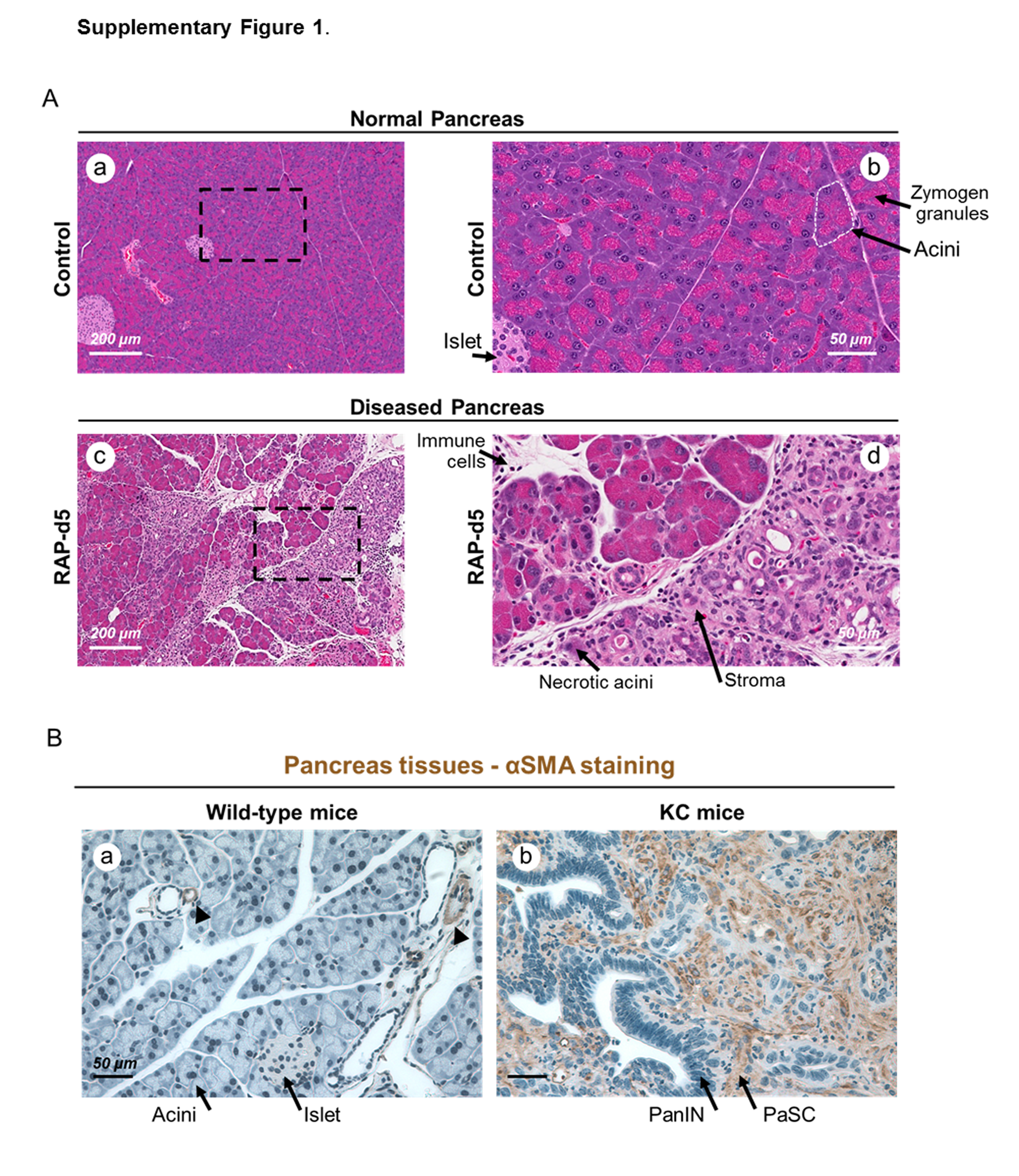

Supplement: SUPPLEMENTARY FIGURE S1 — (A) Pancreas damage in mice during recurrent acute pancreatitis (RAP). C57BL/6 mice were subjected to two episodes of acute cerulein pancreatitis (days 1 and 3; 1 day apart). Each episode consisted of 7 hourly intraperitoneal injections of 50 μg/kg cerulein or saline as vehicle control. Tissues were collected 2 days after the second episode of pancreatitis (day 5; d5). Panels show representative H&E-stained pancreatic tissue sections from mice treated with saline (control; panels a,b) or cerulein (RAP-d5; panels c,d); five to six mice per group. Dotted boxes in panels a,c (scale bar, 200 μm) are shown at higher magnification (scale bar 50 μm) in panels b,d, respectively. As shown in the images, pancreas from control mice (panels a,b) exhibit a normal exocrine parenchyma composed of groups of acinar cells (acini) enriched in digestive enzymes (zymogen granules). Islets (endocrine cells) are also observed in the pictures. At day 5 after RAP (panels c,d), pancreatic tissues display acinar cell necrosis, loss of normal parenchyma, and stromal expansion enriched in immune cells and PaSC. As shown in Figure 1C, α-SMA-positive PaSC were found in RAP-5d but not in control tissues. (B) Pancreas histology in wild type and Kras mice. Representative IHC images showing αSMA staining (brown) in pancreas tissues of 3 month-old wild-type and Ptf1-Cre; LSL-KrasG12D/+ (KC) mice. In wild-type mice, precancerous lesions (pancreatic intraepithelial neoplasia or PanIN) are not present and positive staining is found only in the wall of blood vessels (see arrow head). In KC mice, abundant αSMA-positive cells are found in the stroma surrounding PanINs, indicating activated PaSC. [file Image_1.TIF]

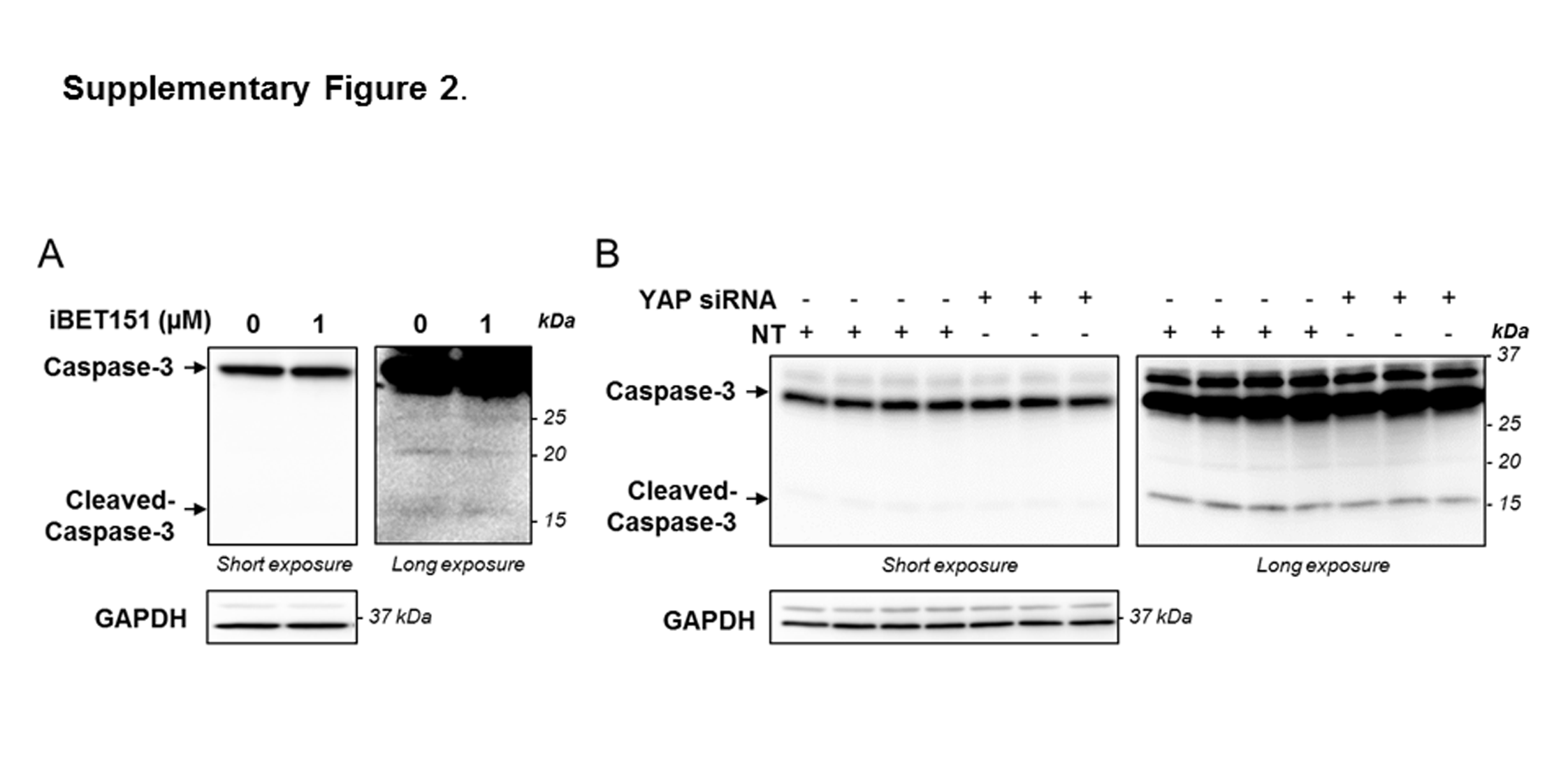

Supplement: SUPPLEMENTARY FIGURE S2 — (A) Immortalized mouse PaSC (imPaSC) were treated with the BET inhibitor, iBET151 for 72 h. (B) imPaSC were transfected with non-targeting negative control (NT, 10 nmol/L) or a targeted siRNA (YAP, 10 nmol/L), and proteins extracted 48 h later. Immunoblots show levels of full length (~35 kDa) and cleaved (~17 kDa) Caspase-3. GAPDH was used as loading control. Compared to controls, neither iBET151 nor YAP siRNA treatments increased levels of cleaved Caspase-3. Data are representative of three to four independent experiments. [file Image_2.TIF]
